# Monitoring ten insect pests in selected orchards in three Azorean Islands: The project CUARENTAGRI

**DOI:** 10.3897/BDJ.11.e100942

**Published:** 2023-03-01

**Authors:** Lucas Lamelas-López, Paulo A. V. Borges, Elisa Tarantino, Maria Manuela Juliano, Jose Carlos Fontes, Cristina Moules, Ricardo Rodrigues, Jessica Machado, José Adriano Mota, Beatriz Sousa, Helder Amaral, Maria da Conceição Filipe, David H. Lopes

**Affiliations:** 1 Centre for Ecology, Evolution and Environmental Changes (cE3c)/Azorean Biodiversity Group, CHANGE – Global Change and Sustainability Institute Faculty of Agricultural Sciences and Environment, University of the Azores, Rua Capitão João d´Ávila, Pico da Urze, Angra do Heroísmo, Azores, Portugal Centre for Ecology, Evolution and Environmental Changes (cE3c)/Azorean Biodiversity Group, CHANGE – Global Change and Sustainability Institute Faculty of Agricultural Sciences and Environment, University of the Azores, Rua Capitão João d´Ávila, Pico da Urze Angra do Heroísmo, Azores Portugal; 2 IUCN SSC Mid-Atlantic Islands Invertebrate Specialist Group, Angra do Heroísmo, Azores, Portugal IUCN SSC Mid-Atlantic Islands Invertebrate Specialist Group Angra do Heroísmo, Azores Portugal; 3 Faculdade de Ciências Agrárias e Ambiente, Universidade dos Açores, Rua Capitão João d’Ávila, São Pedro, 9700-042, Angra do Heroísmo, Azores, Portugal Faculdade de Ciências Agrárias e Ambiente, Universidade dos Açores, Rua Capitão João d’Ávila, São Pedro, 9700-042 Angra do Heroísmo, Azores Portugal; 4 CIBIO-Açores, Centro de investigação em Biodiversidade e Recursos Genéticos, Angra do Heroísmo, Azores, Portugal CIBIO-Açores, Centro de investigação em Biodiversidade e Recursos Genéticos Angra do Heroísmo, Azores Portugal; 5 Serviço de Desenvolvimento Agrário da Terceira, Secretaria Regional de Agricultura e do Desenvolvimento Rural, Direção Regional de Agricultura, Vinha Brava, 9701-880, Angra do Heroísmo, Azores, Portugal Serviço de Desenvolvimento Agrário da Terceira, Secretaria Regional de Agricultura e do Desenvolvimento Rural, Direção Regional de Agricultura, Vinha Brava, 9701-880 Angra do Heroísmo, Azores Portugal; 6 FRUTER – Associação de Produtores de Frutas, de Produtos Hortícolas e Florícolas da Ilha Terceira, Angra do Heroísmo, Azores, Portugal FRUTER – Associação de Produtores de Frutas, de Produtos Hortícolas e Florícolas da Ilha Terceira Angra do Heroísmo, Azores Portugal; 7 Serviço de Desenvolvimento Agrário de São Jorge, Secretaria Regional dae Agricultura e do Desenvolvimento Rural, São Jorge, 9800-423, Urzelina, Azores, Portugal Serviço de Desenvolvimento Agrário de São Jorge, Secretaria Regional dae Agricultura e do Desenvolvimento Rural, São Jorge, 9800-423 Urzelina, Azores Portugal; 8 Direção de Serviços de Agricultura, Direção Regional de Agricultura, Secretaria Regional de Agricultura e do Desenvolvimento Rural, Quinta de São Gonçalo, 9500-343, Ponta Delgada, Azores, Portugal Direção de Serviços de Agricultura, Direção Regional de Agricultura, Secretaria Regional de Agricultura e do Desenvolvimento Rural, Quinta de São Gonçalo, 9500-343 Ponta Delgada, Azores Portugal

**Keywords:** agriculture, dataset, invertebrates, Macaronesia, orchards, pest risk, pheromone traps

## Abstract

**Background:**

The data we present are part of the CUARENTAGRI project, which involves all archipelagos of the Macaronesia (Azores, Madeira, Canary Islands and Cabo Verde). The project aims to: i) identify and evaluate the risks associated with the introduction of new arthropod pests; ii) study the population dynamics of selected arthropod pest species currently responsible for the damage of key target crops and iii) develop monitoring systems, based on prediction and/or population dynamics of the crop pests, creating warnings and a phytosanitary prevention system. In this contribution, we compile data for three Azorean Islands (Terceira, São Jorge and São Miguel Islands), where pheromone-baited traps were placed in pastures, potato fields and several orchards’ types (apples, banana, chestnuts, olives, orange and strawberry), during three consecutive years (2020, 2021 and 2022).

**New information:**

A total of 114,827 specimens of insects (Arthropoda, Insecta) were collected, belonging to four orders, six families and ten recorded pest species. A total of eight species are considered introduced (*Cosmopolitessordidus* (Germar, 1824), *Drosophilasuzukii* (Matsumura, 1931), *Bactroceraoleae* (Rossi, 1790), *Ceratitiscapitata* (Wiedemann, 1824), *Phthorimaeaoperculella* (Zeller, 1873), *Cydiapomonella* (Linnaeus, 1758), *Cydiasplendana* (Hübner, 1799) and *Grapholitamolesta* (Busck, 1916); n = 84,986 specimens) and two native non-endemic (*Mythimnaunipuncta* (Haworth, 1809) and *Spodopteralittoralis* (Boisduval, 1833); n = 17,465 specimens). This study intended to contribute to a better knowledge of the arthropods pests that can affect the Azorean crops and will serve as a baseline for future monitoring actions, pest risk assessments and prevention systems.

## Introduction

Crop pests represent a worldwide threat to food security, leading to substantial agricultural production losses (Flood 2010, Fisher et al. 2012, Bebber et al. 2014). Although pathogens, weeds and several groups of vertebrate and invertebrate are responsible for damage in crops, arthropods are probably one of the main threats at global scale to agroecosystems, through direct impacts as well as disease transmission to plants (e.g. Douglas (2018)). However, arthropods can also have a positive effect in the crops, by acting, for example, as natural enemies of pest species (e.g. Ameixa et al. (2018), Ferrante et al. (2022)). For instance, Costanza et al. (1997) estimate that the economic value of the arthropod generalist predators is around $400 billion per year.

In the Macaronesian Islands, the unique climatic conditions allow the cultivation of crops not possible in the European mainland. In addition to the favourable climatic conditions, the landscape fragmentation, the high touristic activities and the increasing commercial activities make these Islands easily subjected to invasion by exotic species and harmful organisms (Borges et al. 2020).

The risk associated with the accidental introduction of exotic arthropods is very high (see e.g. Borges et al. (2013), Borges et al. (2022a)), some of them with potential pest status. The intergovernmental organisation responsible for cooperation in plant health within the Euro-Mediterranean region (EPPO), in collaboration with other international partners, conducts pest risk analyses for the Mediterranean area and Central Europe. Despite past changes in regulations actions, with the production of a large number of Standards in the areas of plant protection products and plant quarantine, the current situation still remains poorly assessed for the Macaronesian Islands.

The project CUARENTAGRI aims to contribute to prevent and/or reduce the arrival, establishment and proliferation of new harmful organisms to the Macaronesian Islands. To achieve such a goal, there is the need to promote better training in pest risk analysis (PRA) for technicians and enhance the dissemination of appropriate information to farmers and citizens in general.

Currently, it is very important to know which harmful organisms from the European Union priority lists are most likely to be introduced in Macaronesia, facilitating the delineation, modelling and development of contingency plans in advance to deal with these phytosanitary problems.

Relative to the monitoring of the population dynamics of the main crop pests, different types of traps and attractants have been used in the field to allow the detection of their arrival, creating an alert system and making the emission of agricultural warnings. Phytosanitary sheets were made fortnightly in the three targeted Azorean Islands (Terceira, São Jorge and São Miguel), with information on the arrival and population dynamics of the key pests in the economically most important crops. In addition, some activities of information dissemination and awareness-raising were developed for farmers in the field. The data we present are part of the CUARENTAGRI project (see https://www.cuarentagri.com/ September 2019 - August 2022), which involves the outermost regions of the European Union of Azores, Madeira, Canary Islands and Cabo Verde and Senegal as third countries.

## General description

### Purpose

To provide an arthropod pests inventory in the Azores Archipelago, using three islands as study case (Terceira, São Jorge and São Miguel), based on data collected in several types of orchards and crops. We aim to include identification of arthropod pests responsible by damaging crops, evaluate the risk associated with their introduction and to develop monitoring systems, based on prediction and/or population dynamics and phenology of the crop pests. This study aims to contribute to a better knowledge of the arthropods pests that can affect the Azorean crops and will serve as a baseline for future monitoring actions, pest risk assessments and impacts warning and prevention systems.

### Additional information

The data we present are part of the CUARENTAGRI project, which involves the archipelagos of the Macaronesia region: Azores, Madeira, Canary Islands and Cabo Verde. In this publication, we present all data related to the Azores Archipelago, collected during three consecutive years (2020, 2021, 2022). The CUARENTAGRI project includes additional objectives, as for example, the creation of an alert network with the scope to carry on an early detection pest plan and specific training activities for technicians in plant protection sector. Specific details of the original project can be consulted in the website of the project (https://www.cuarentagri.com/).

## Project description

### Title

Inventory of Arthropod pests in Azorean orchards: The project Cuarentagri

### Personnel

Project leaders: David Horta Lopes (UAç), José Asterio Guerra (GMR-Canárias), Miguel Angelo Carvalho (UMa), Luis Dantas (DRAM), Raimundo Cabrera (ULL) and Estrela Hernandez (ICIA).

Team members: José Carlos Goulart Fontes (UAç), Maria Manuela Juliano (UAç), Elisa Tarantino (UAç), Cristina Moules (SDAT/SRADR), Ricardo Rodrigues (FRUTER), José Adriano Mota (DRADSA/DRSAg/SRADR), Jéssica Machado (SDASJ/SRADR), Beatriz Sousa (DSA/DRAgDRA/DSA/SRADR), Helder Amaral (DSA/DRAgDRA/DSA/SRADR), Maria da Conceição Filipe (FRUTER) and António Lopes (DSA/DRAgDRA/DSA/SRADR).

External Consultants: António Maria Marques Mexia (ISA/UL).

Taxonomists: Paulo A.V. Borges (UAç) and Estrela Hernandez (ICIA).

Darwin Core Database management: Lucas Lamelas-López and Paulo A.V. Borges (UAç).

### Study area description

The study comprises three islands of the Azores Archipelago, which is located in the northern Atlantic Ocean (roughly at 38°43'17''N, 27°13'14''W) and is formed by nine islands of volcanic origin and several small islets. The Archipelago is divided into three main groups: The Western Group (Flores and Corvo), the Central Group (Faial, Pico, São Jorge, Graciosa and Terceira) and the Eastern Group (São Miguel and Santa Maria). The studied islands were Terceira (area: 402 km^2^, 1023 m a.s.l.), São Jorge (area: 246 km^2^, 1053 m a.s.l.) and São Miguel (757 km^2^, 1103 m a.s.l.). The climate of the Archipelago is temperate oceanic, characterised by regular and abundant precipitation, high levels of air relative humidity and persistent winds, mainly during the winter season. The land-use of the Azores is dominated by several types of agroecosystems. The main agricultural habitats are semi-natural pastures at high elevation (500-700 m a.s.l.), intensive pastures and maize fields (in rotation with intensive pastures in the summer), mostly between 0 and 500 m a.s.l. Orchards and vineyards are also present and restricted to low elevations near coastal areas in some microclimatic ideal conditions for each target fruit tree.

### Design description

The sampled agroecosystems were selected by investigators of the University of the Azores in cooperation with the technicians from the Regional Secretariat for Agriculture and Rural Development and FRUTER Producers Cooperative of Terceira Island and included several types of orchards and crops, as potato fields, apples, banana, chestnuts, olives, orange and strawberry and pastures.

The sampling methods included the installation of traps baited with pheromones, using different attractant types. The traps remain active during all year or only for few months, according to pest life cycles. The traps were monitored regularly to replace the attractant or pheromone. The collected specimens were identified by an expert taxonomist in the laboratory. The study was conducted during three consecutive years (2020, 2021 and 2022).

### Funding

This work was financed under the project CUARENTAGRI by Cooperation Programs INTERREG V A (Spain-Portugal) and MAC 2014-2020. Darwin Core Database management was funded by the Project project FCT-UIDB/00329/2020-2024 (Thematic Line 1 – integrated ecological assessment of environmental change on biodiversity) and Portal da Biodiversidade dos Açores (2022-2023) - PO Azores Project - M1.1.A/INFRAEST CIENT/001/2022.

## Sampling methods

### Study extent

The study was conducted in three islands of the Azores Archipelago, Terceira, São Jorge and São Miguel. The sampled agricultural areas included several crops and orchards types, as potato fields, apples, banana, chestnuts, olives, orange and strawberry and pastures.

### Sampling description

The sampling methods included the installation of three traps per each plot baited with pheromones, using different attractant types. Most of the traps were commercial with the food attractant (e.g. *Ceratitiscapitata* (Wiedemann, 1824)), sexual pheromone (e.g. *Bactroceraoleae* (Rossi, 1790) or *Phthorimaeaoperculella* (Zeller, 1873)) or aggregation pheromones (e.g. *Cosmopolitessordidus* (Germar, 1824)) depending of the type of pest.

The traps were installed in each orchard in three diferent sites with the same plant host during the períod of normal appearance of the adults of the different pests sometimes only for few months, depending of the pest life cycle and fruits in the host and in the case of the most economomically important key pests during the whole year (*C.capitata*, *C.sordidus*, thrips). The traps were monitored every two weeks in the field and the collected captured adults were identified by an expert taxonomist in the laboratory, determining the individual sex when possible and registered in the web platform of the project. The sampling protocol was implemented during three consecutive years (2020, 2021 and 2022). Additionally, phytosanitary sheet reports were regularly elaborated and provided to technicians and farmers in order to inform them about pests identity, their abundance and spread status.

### Quality control

All collected individuals were identified by expert taxonomists in the laboratory. When possible, the sex of the individuals was provided. The taxonomic nomenclature follows the most recent checklist of Azorean arthropopds (Borges et al. 2022b).

### Step description

The sampled agroecosystems were selected by investigators of the University of the Azores in cooperation with the technicians from the Regional Secretariat for Agriculture and Rural Development and FRUTER Producers Cooperative of Terceira Island, which included several types of crops and orchards. Pheromone-baited traps were used to sample the arthropod pests, which remain deployed during the appearance of the adults of the different pests, sometimes during all year (*C.capitata*, *C.sordidus*, trips) and sometimes only for few months, depending of the pest life cycle and monitored in one or two weeks periods. The sampling protocol comprise three consecutive years (2020, 2021 and 2022). The collected individuals were identified by expert taxonomists in the laboratory. Additionally, phytosanitary sheet reports to technicians and farmers were regularly elaborated, including information about pests identity, their abundance and spread status.

## Geographic coverage

### Description

The study was conducted on Terceira, São Jorge and São Miguel Islands, Azores, Portugal.

### Coordinates

37.697 and 38.812 Latitude; -28.339 and -25.117 Longitude.

## Taxonomic coverage

### Description

The following orders of Insecta Class are covered: Thysanoptera, Hemiptera, Coleoptera, Lepidoptera, Diptera.

### Taxa included

**Table:** 

Rank	Scientific Name	Common Name
phylum	Arthropoda	Arthropods
class	Insecta	Insects
order	Coleoptera	Beetles
order	Hemiptera	Bugs
order	Lepidoptera	Butterflies and Moths
order	Diptera	Flies
order	Thysanoptera	Thrips

## Temporal coverage

**Data range:** 2020-1-01 – 2022-10-28.

## Usage licence

### Usage licence

Other

### IP rights notes

Creative Commons Attribution Non Commercial (CC-BY-NC) 4.0 License

## Data resources

### Data package title

Inventory of Arthropod pests in Azorean orchards: The project CUARENTAGRI

### Resource link

https://www.gbif.org/dataset/8f856bf9-dcb6-4154-93c5-ae84fd423a47

### Alternative identifiers

http://ipt.gbif.pt/ipt/resource?r=cuarentagri_azores_2022

### Number of data sets

2

### Data set 1.

#### Data set name

Event Table

#### Data format

Darwin Core Archive

#### Character set

UTF-8

#### Download URL

http://ipt.gbif.pt/ipt/resource?r=cuarentagri_azores_2022

#### Data format version

version 1.6

#### Description

The dataset is available on the Global Biodiversity Information Facility platform, GBIF (Lamelas-López et al. 2022). The following data table includes all the records for which a taxonomic identification of the species was possible. The dataset submitted to GBIF is structured as a sample event dataset, with two tables: event (as core) and occurrences (abundance data). The data in this sampling event resource have been published as a Darwin Core Archive (DwCA), which is a standardised format for sharing biodiversity data as a set of one or more data tables. The core data file contains 409 records (eventID). This IPT (Integrated Publishing Toolkit) archives the data and, thus, serves as the data repository. The data and resource metadata are available for download from Lamelas-López et al. (2022).

**Data set 1.** 

Column label	Column description
eventID	Identifier of the events, unique for the dataset.
stateProvince	Name of the region of the sampling site.
islandGroup	Name of archipelago.
island	Name of the island.
country	Country of the sampling site.
countryCode	ISO code of the country of the sampling site.
locality	Locality of the sampling site.
locationRemarks	Additional information about the locality.
decimalLongitude	The geographic longitude (in decimal degrees, using the spatial reference system given in geodeticDatum) of the geographic centre of a Location.
decimalLatitude	The geographic latitude (in decimal degrees, using the spatial reference system given in geodeticDatum) of the geographic centre of a Location.
geodeticDatum	The ellipsoid, geodetic datum or spatial reference system (SRS) upon which the geographic coordinates given in decimalLatitude and decimalLongitude are based.
coordinateUncertaintyInMeters	Uncertainty of the coordinates of the centre of the sampling plot in meters.
coordinatePrecision	A decimal representation of the precision of the coordinates given in the decimalLatitude and decimalLongitude.
georeferenceSources	A list (concatenated and separated) of maps, gazetteers or other resources used to georeference the Location, described specifically enough to allow anyone in the future to use the same resources.
locationID	Identifier of the location.
samplingProtocol	The sampling protocol used to capture the species.
sampleSizeValue	The numeric amount of time spent in each sampling.
sampleSizeUnit	The unit of the sample size value.
day	Day of the event.
month	Month of the event.
year	Year of the event.
eventDate	Date or date range the record was collected.
habitat	The habitat of the sampling site.

### Data set 2.

#### Data set name

Occurrence Table

#### Data format

Darwin Core Archive

#### Character set

UTF-8

#### Download URL

http://ipt.gbif.pt/ipt/resource?r=cuarentagri_azores_2022

#### Data format version

version 1.6

#### Description

The dataset is available on the Global Biodiversity Information Facility platform, GBIF (Lamelas-López et al. 2022). The following data table includes all the records for which a taxonomic identification of the species was possible. The dataset submitted to GBIF is structured as a sample event dataset, with two tables: event (as core) and occurrences (abundance data). The data in this sampling event resource have been published as a Darwin Core Archive (DwCA), which is a standardised format for sharing biodiversity data as a set of one or more data tables. The core data file contains 412 records (occurrenceID). This IPT (Integrated Publishing Toolkit) archives the data and, thus, serves as the data repository. The data and resource metadata are available for download from Lamelas-López et al. (2022).

**Data set 2.** 

Column label	Column description
eventID	Identifier of the events, unique for the dataset.
type	Type of the record, as defined by the Public Core standard.
licence	Reference to the licence under which the record is published.
institutionID	The identity of the institution publishing the data.
institutionCode	The code of the institution publishing the data.
datasetName	Name of the dataset.
basisOfRecord	The nature of the data record.
occurrenceID	Identifier of the record, coded as a global unique identifier.
recordedBy	A list (concatenated and separated) of names of people, groups or organisations who performed the sampling in the field.
identifiedBy	A list (concatenated and separated) of names of people, groups or organisations who performed the sampling in the field.
dateIdentified	The date on which the subject was determined as representing the Taxon.
organismQuantity	A number or enumeration value for the quantity of organisms.
organismQuantityType	The type of quantification system used for the quantity of organisms.
lifeStage	The life stage of the organisms captured.
scientificName	Complete scientific name including author and year.
kingdom	Kingdom name.
phylum	Phylum name.
class	Class name.
order	Order name.
family	Family name.
genus	Genus name.
specificEpithet	Specific epithet.
scientificNameAuthorship	Name of the author of the lowest taxon rank included in the record.
taxonRank	Lowest taxonomic rank of the record.
establishmentMeans	The process of establishment of the species in the location, using a controlled vocabulary: 'native', 'introduced', 'endemic', 'indeterminate'.

## Additional information

**Results**

We collected a total of 114,827 specimens of insects, belonging to four orders, six families and ten species. A total of eight species are considered introduced (n = 84,986 specimens) and two native non-endemic (n = 17,465 specimens). No endemic species were recorded and 12,376 specimens were identified at order level, all belonging to Thysanoptera (Table 1).

In general, the most abundant pest species was the banana root borer *C.sordidus* (Coleoptera, Curculionidae) (n = 45,759), which was mainly captured in Terceira Island (n = 35,412) and the spotted wing drosophila *Drosophilasuzukii* (Matsumura, 1931) (Diptera, Drosophilidae) (n = 25,592), recorded mainly in São Miguel Island (n = 21,390). The family Tortricidae (Lepidoptera) included the species with lowest abundance in the orchards, *Cydiasplendana* (Hübner, 1799) (n = 411); *C.pomonella* (Linnaeus, 1758) (n = 101); and *Grapholitamolesta* (Busck, 1916) (n = 40; Table 1).

Although most of detected pest species are considered introduced, two species of Noctuidae family (Lepidoptera) are considered native non-endemic species: *Spodopteralittoralis* (Boisduval, 1833) (n = 17,204) and *Mythimnaunipuncta* (Haworth, 1809) (n = 261; Table 1).

The island with highest captures of pest species was Terceira with 64,685 captured individuals; São Miguel recorded a total of 49,310 individuals and, finally, São Jorge, with significantly less captures (n = 841), mainly associated with the low sampling effort in the Island.

Banana orchards and mixed citrus recorded the highest abundance of pest species. In the case of banana orchards mainly by the presence of the banana root borer *C.sordidus* (around 78% of total captures) and individuals of the Thysanoptera order. In the mixed citrus orchards, mainly two species of Diptera, *C.capitata* and *D.suzukii* (88% of total captures) were recorded (Table 2). This is mainly due to the climatic conditions of those areas and also to the abundance of food to these pests, namely banana roots and recently cut banana plants in the soil of the banana orchards and a large mixture of matured fruits in the citrus orchards.

Moreover, some studied orchards recorded less than 50 individuals of pest species, as fig tree orchard (n = 46 individuals), tangerine tree orchard (n = 35) and lemon tree orchard (n = 6; Table 2). These orchards, as well plum trees, seem to be more resistant to pest species, due to the extensive use of pesticides and also due to less abundance of the other fruits more attractive on maturation period of these fruits.

The most widespread pest species were the Diptera *C.capitata* and *D.suzukii*, which were detected at more than 50% of the studied orchards types. This is according to another studies (e.g. Pimentel et al. (2016), Pimentel et al. (2017)).

As expected, Thysanoptera were only detected in banana orchards, *B.oleae* only in olive tree orchards and *C.pomonella* in apple tree orchards (Table 2).

**Pests information and Conclusion**

The ten species of agricultural pests recorded and the known impacts are summarily described as follows:

*Cosmopolitessordidus* (Germar, 1824) is one of the most important insect pests of banana orchards worldwide, producing different types of impacts during the life of the crop, namely destroying the roots and plants and making the banana plants susceptible to fall at the end of their production cycle (Gold et al. 2001). Consequently, in the Azores, this species was the main pest species recorded in banana orchards, although it was also detected in apple tree orchards (Lopes et al. 2008, Lopes et al. 2012).

*Drosophilasuzukii* (Matsumura, 1931) is a major pest species in European agricultural systems, affecting several types of orchards and leading to considerable losses in fruit production (e.g. Mortelmans et al. (2012), Lengyel et al. (2015), Japoshvili et al. (2018)). In the Azores, this species was recorded in several types of orchards (e.g. apple tree, blueberry, strawberry, strawberry-araça, coffee, fig tree, mixed citrus and orange tree orchards) and causes some important damage mainly in the strawberry parcels where it is relatively abundant (Melo et al. 2019).

*Bactroceraoleae* (Rossi, 1790) is probably one of the main pest species of olive trees in the world (Hladnik 2017, Müller et al. 2019). The main impacts consist of fruit damaging and, consequently, loss of commercial value. In the Azores, this species was abundant in olive tree orchards, being the main pest species on this type of agroecosystem and causing production losses near 80% for the orchards without phytosanitary treatment (Lopes et al. 2010, Lopes et al. 2011, Moules et al. 2022).

*Ceratitiscapitata* (Wiedemann, 1824) can have an impact in several types of crops, mainly by attacking soft fruits, causing severe economic losses (De Meyer et al. 2008). This species was recorded in diverse types of Azorean orchards and also with large populations in coffee plants. It causes the early fall of the fruits before their maturation is completed in most fruit orchards (Lopes et al. 2021).

*Phthorimaeaoperculella* (Zeller, 1873) is one of the major pest species that affect potato crops. Its impacts are produced by its larvae mainly during storage process and also in the production fields (Niroula and Vaidya 2004). In the Azores, this was the unique species recorded on potato crops and also on those stored.

*Mythimnaunipuncta* (Haworth, 1809) is responsible for economic damage in Poaceae cultivars (Mendes and Lopes 2005, Madruga et al. 2022). In the Azores, this species nowadays appears on the pastures in low densities when compared with *S.littoralis* that every year increases its populations densities on the Azorean pastures causing important economic losses.

*Spodopteralittoralis* (Boisduval, 1833) is one of the major lepidopteran pests in many regions of the world, impacting on several types of agricultural areas, such as grasslands, gardens or fruit tree plantations (Lopez-Vaamonde 2010). In the Azores, this species is currently the main pest of pastures and some vegetable gardens and horticultural cultures starting to affect lettuce and other leaf cultures.

*Cydiapomonella* (Linnaeus, 1758) is one of the most important pests on apple tree orchards worldwide, due to the damages in the fruits, which lead to economic losses in production process (Mátray and Herz 2021). In the Azores, this species was detected on apple tree orchards affecting and destroying mainly the fruits before harvest (Lopes et al. 2008), but their abundance is lower compared with other pests, such as *C.capitata*.

*Cydiasplendana* (Hübner, 1799) is a major pest of chestnut orchards, affecting the fruits by destroying them and causing very important economic losses for the chestnut producers. This species was already highlighted as an important pest of chestnut orchards in the Azores (Lopes et al. 2007, Lopes et al. 2008) (see Table 2).

*Grapholitamolesta* (Busck, 1916) is probably one of the major pest species that affect fruit trees (e.g. peach and apple trees) in several regions of the world (Strapasson et al. 2016). In the Azores, this species is probably a minor pest, given its low abundance and limited distribution mainly on the peach, plum and apple orchards where it may cause the total destruction of the fruit (Lopes et al. 2008).

The results of this publication contribute to a better knowledge of the arthropods pests that can affect the Azorean crops. For example, *C.capitata* and *D.suzukii* demonstrated to be widespread and abundant pest species across several agroecosystems types and *C.sordidus* is probably the most important pest species on banana orchards. Although most of the pest species are introduced, some are native due to their high dispersal ability. This is the case of *M.unipuncta* and *S.littoralis* that are major pests in intensive pastures and maize fields and now are starting to affect horticultural cultures. These results also will serve as a potential baseline for future monitoring actions, pest risk assessments and prevention systems.

## Figures and Tables

**Table 1.** Inventory of the identified pest species recorded in Azorean agroecosystems, in São Jorge (SJG), Terceira (TER) and São Miguel (SMG) Islands, between 2020 and 2022. The colonisation status (Status) and abundance values are provided.

Order	Family	Species	Colonisation status	Abundance per island		
				SJG	SMG	TER
Coleoptera	Dryophthoridae	*Cosmopolitessordidus* (Germar, 1824)	introduced	0	10347	35412
Diptera	Drosophilidae	*Drosophilasuzukii* (Matsumura, 1931)	introduced	383	21390	3819
Diptera	Tephritidae	*Bactroceraoleae* (Rossi, 1790)	introduced	0	0	3246
Diptera	Tephritidae	*Ceratitiscapitata* (Wiedemann, 1824)	introduced	458	985	4214
Lepidoptera	Gelechiidae	*Phthorimaeaoperculella* (Zeller, 1873)	introduced	0	4010	179
Lepidoptera	Noctuidae	*Mythimnaunipuncta* (Haworth, 1809)	native	0	63	198
Lepidoptera	Noctuidae	*Spodopteralittoralis* (Boisduval, 1833)	native	0	5079	12125
Lepidoptera	Tortricidae	*Cydiapomonella* (Linnaeus, 1758)	introduced	0	3	98
Lepidoptera	Tortricidae	*Cydiasplendana* (Hübner, 1799)	introduced	0	0	411
Lepidoptera	Tortricidae	*Grapholitamolesta* (Busck, 1916)	introduced	0	0	40
Thysanoptera		Thysanoptera		0	7433	4943

**Table 2.** Abundance of pest species of Insecta Class per agroecosystem type. The presented data aggregate the records obtained in the three studied islands (São Jorge, Terceira and São Miguel).

	Coleoptera	Diptera			Lepidoptera					
Agroecosystem	*C.sordidus*	*B.oleae*	*C.capitata*	*D.suzukii*	*C.pomonella*	*C.splendana*	*G.molesta*	*M.unipuncta*	*P.operculella*	*S.littoralis*
Apple tree orchard	143	0	636	27	101	21	0	0	0	0
Banana orchard	45616	0	46	0	0	0	0	0	0	0
Blueberry orchard	0	0	16	244	0	0	0	0	0	0
Chestnut orchard	0	0	0	0	0	390	31	0	0	0
Coffee plantation	0	0	1799	14	0	0	0	0	0	0
Fig tree orchard	0	0	0	46	0	0	0	0	0	0
Lemon tree orchard	0	0	6	0	0	0	0	0	0	0
Mixed citrus	0	0	2695	21411	0	0	0	0	0	0
Olive tree orchard	0	3246	0	0	0	0	0	0	0	0
Orange tree orchard	0	0	191	2858	0	0	0	0	0	0
Pasture	0	0	0	0	0	0	0	261	0	16148
Plum tree orchard	0	0	87	0	0	0	9	0	0	0
Potato culture	0	0	0	0	0	0	0	0	4189	0
Several cultures	0	0	146	88	0	0	0	0	0	0
Strawberry orchard	0	0	0	480	0	0	0	0	0	0
Strawberry-Araçá orchard	0	0	0	249	0	0	0	0	0	0
Tangerine tree orchard	0	0	35	0	0	0	0	0	0	0
Vegetable garden	0	0	0	0	0	0	0	0	0	993
Vineyard	0	0	0	175	0	0	0	0	0	63
